# Editorial

**Published:** 1854-05

**Authors:** 


### THE MEDICAL EXAMINER.

### PHILADELPHIA, MAY, 1854.

In our next issue, we shall publish the proceedings of the present session of the American Medical Association. It will be remembered by our readers, that a number of amendments to the Constitution will be presented for its consideration. Its action on these, and the Report on a plan for organization of state and local societies—a measure fraught with the highest consequences to the well-being of the profession—are anticipated with great anxiety.

We believe that in future times, the formation of our National Association will be regarded as the most important epoch in the history of medicine in our country. It has already effected much good in stimulating and elevating the American medical mind. We know that the French Institute, the British Association, the Medico-Chirurgical Society of England, and others that we might mention, have all exercised an immense and continuous influence in their respective spheres, and we have a right to expect that a similar effect will follow our own organization.

To take our proper station in the commonwealth of science, we need hardly say that a higher literature than we have ever yet exhibited, is absolutely necessary—a literature which shall be, in some measure, the exponent of that mental activity and spirit of investigation which is so characteristic of the American people.

May we not expect, judging from the value of its Transactions already published, that our own Association will greatly tend to form and rapidly advance such a desirable result?

---

In our April number, under the head of Medical News, we inserted an article from the Buffalo Medical Journal, giving an account of the proceedings of the State Medical Society of New York. In this, the New York Medical College was stated to be in Fourteenth Street, and the University in Thirteenth Street—their locations should have been reversed.

